# A slab window in the south rim of the Parece-Vela Basin

**DOI:** 10.1038/s41598-025-86913-z

**Published:** 2025-01-18

**Authors:** Zhang Zhen, Xu dong, Zhao Lintao

**Affiliations:** 1 Fourth Institute of Oceanography, Ministry of Natural Resources, Beihai, 536000 China; 2 Second Institute of Oceanography, Key Laboratory of Submarine Geosciences, Ministry of Natural Resources, Hangzhou, 310000 China

**Keywords:** Slab Window, Seamounts, Mariana Trench, Parece-Vela Basin, Geochemistry, Tectonics

## Abstract

Slab windows represent regions within the mantle that are largely devoid of slab material, facilitating direct communication between the mantle above and below the subducting slab. This unprecedented interaction disrupts the conventional material-energy exchange mechanisms between the subducted slab and mantle wedge, giving rise to anomalous heat flow, distinct magmatism, metamorphism, and geophysical features. Geochemical analyses of samples collected from the southern margin of the Parece-Vela Basin have illuminated the magmatic processes associated with a slab window. C20-3 is indicative of a hot and dry mantle upwelling that occurs through the slab window. On the other hand, C20-1 signifies an adakitic rock, which originates from the melting of oceanic crust at the periphery of the slab window. Slightly east of the slab window, C21-1 reveals evidence of a heated depleted mantle, influenced by the hot and dry mantle upwelling through the slab window. Additionally, a common Island Arc Basalt (IAB), denoted as C21-2, was also observed in the island arc region. Topographical data highlights the irregular distribution of numerous seamounts between the West Mariana Ridge and the Parece-Vela Basin spreading center. Seismological records reveal a preponderance of strike-slip earthquakes in the southern seamount region, alongside exceptionally high heat flow measurements at the northwest extremity of this area. The shape of the slab window can be roughly modeled by analyzing the distribution of earthquakes. Collectively, these observations lead us to postulate the existence of a slab window beneath the southern rim of the Parece-Vela Basin, likely attributed to the tearing of the subducting Pacific Plate along the strike-slip fault situated between the Ulithi and Fais Atolls.

## Introduction

The “slab window,” a geological phenomenon referring to regions in the mantle beneath convergent plate margins where the subducting slab is largely absent due to either the subduction of spreading centers or the tearing of the subducted slab, has garnered significant attention in recent studies^1^. The concept was initially formulated based on observations of subducted spreading centers in Japan, the Aleutian Islands, and Sumatra^2–4^. Subsequent research advanced this understanding, proposing that fragile young slabs can tear during subduction, thereby creating slab windows^5–7^. For example, Chen, et al.^8^proposed that the varying angles of the Indian Plate’s northward subduction result in the tearing of the subducting plate, ultimately leading to the slab windows. Distinct from traditional subduction zones, slab windows enable direct interactions between sub-slab mantle and above-slab mantle. This unprecedented interaction modifies the conventional material-energy exchange processes between the slab and mantle wedge^9^, resulting in a suite of anomalous geological features. These include unusually high heat flow, unique magmatism, metamorphism, distinct geomorphological patterns, enhanced fluid activity^1^, low seismic wave velocity^10^, missing Benioff zone^11^, and changes in stress field^7^ within the overlying plate above the slab window.

In the southern rim of the Parece-Vela Basin, a scattered distribution of intraplate seamounts is observed. This region is characterized by a notably lower frequency of earthquakes compared to adjacent areas, and the earthquakes that do occur are predominantly of the shearing type, contrasting with the more typical thrust earthquakes associated with subducted slabs. Additionally, geological evidence indicates that the subducted slab in this region has been transected by a north-south trending shearing fault, which penetrates through the lithosphere^12^. Based on these observations, we hypothesize that the shearing fault may have created a slab window beneath this region, contributing to the formation of the seamounts and the occurrence of uncommon earthquake types. To investigate the presence of a slab window, we conducted a major elements and trace elements analysis of several samples collected from the southern rim of the Parece-Vela Basin, aiming to elucidate the magma processes related to the slab window. Furthermore, we utilized earthquake distribution data to develop a preliminary model of the slab window’s shape.

## Geological setting

**Fig. 1:**
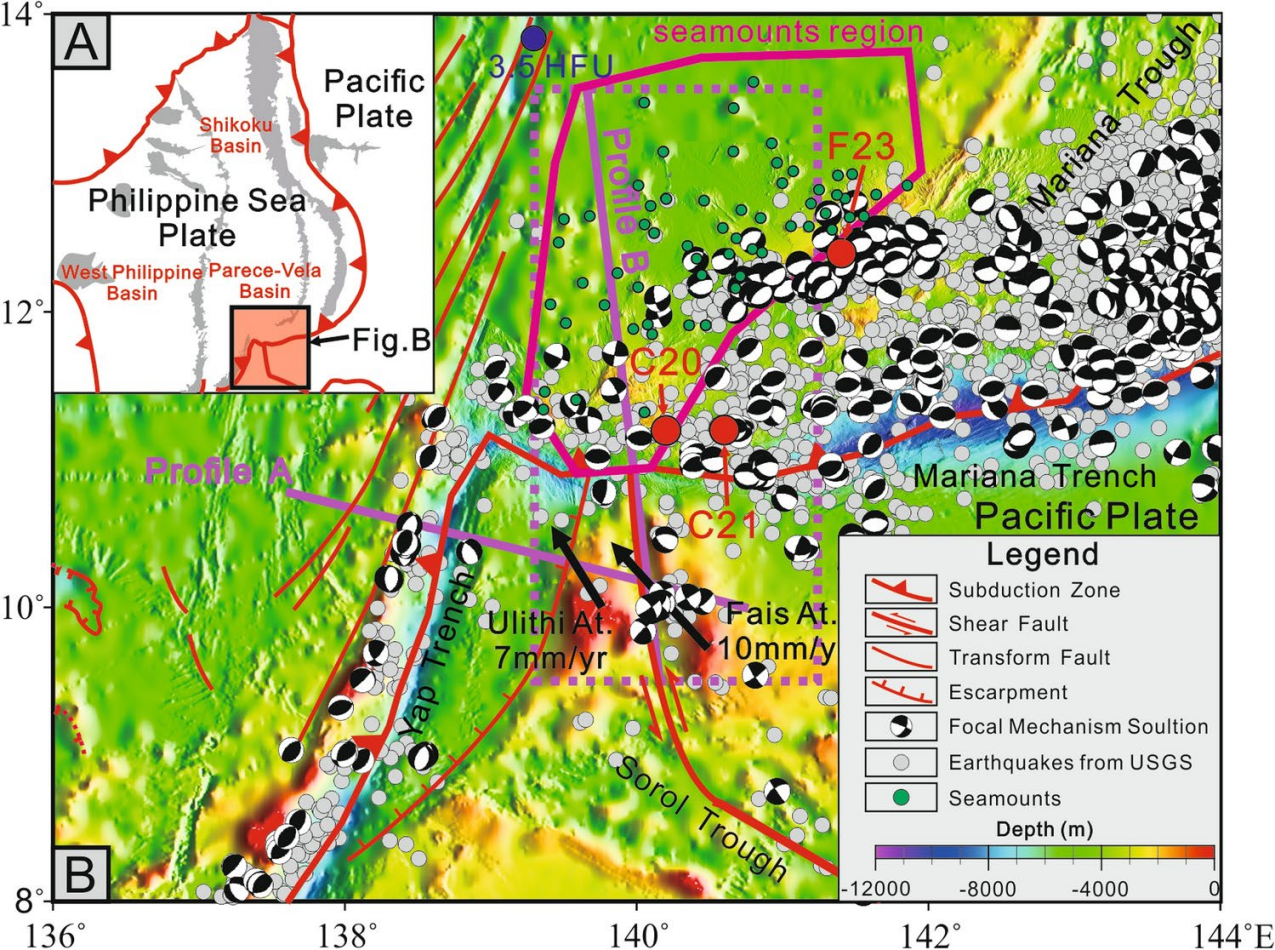
Geologic map of south rim of Parece-Vela Basin with focal mechanism solution from CMT (1976–2023, M>5), earthquakes from USGS (1976–2023, M>2.5) and sample locations (red dots). Black arrows at Ulith Atoll and Fais Atoll show the GPS velocity data from Lee^16^. C20 is situated at the edge of a small plateau, C21 is positioned alongside several relatively small seamounts, F23 is located in the southern segment of the Mariana Trough. (Created by GMT 5.1.1 sofware; https://www.earthbyte.org/gmt-version-5-2-released/).

The Parece-Vela Basin serves as a paradigmatic example of a back-arc basin^13^. Along its southern rim, an array of seamounts is conspicuously situated, as depicted in Figure 1. These seamounts span the region bounded by the West Mariana Ridge and the spreading center of the Parece-Vela Basin, forming a distinctive landscape. This seamount region is positioned at a convergent plate margin, where the Pacific Plate and the Caroline Plate subduct beneath the Philippine Sea Plate along the Mariana Trench and the Yap Trench to the north. According to plate tectonic principles, thrusting earthquakes associated with subduction processes would typically dominate this setting. Nevertheless, strike-slip earthquakes are surprisingly more prevalent compared to thrusting earthquakes (Fig. 1) in the southern segment of the seamount region, which is an uncommon feature in typical subduction zones. To the southeast of the seamount cluster, an eastward-trending segment of the Mariana Trough spreading center stands apart, exhibiting a marked contrast to the overall north-south orientation of the Mariana Trough spreading center^14,15^. Pearce, et al.^15^ classified this anomalous spreading center segment as lithosphere based on geochemical data. Notably, the frequent occurrence of tension earthquakes in this area indicates that this segment experiences active extension.

The southern flank of the Mariana Trench hosts the Pacific and Caroline Plates, with the Sorol Trough, extending towards the Yap Trench, commonly regarded as the demarcation line between these two plates^16^. Zhang, et al.^12^proposed a strike-slip fault between the Ulithi and Fais Atolls, identifying it as the plate boundary with earthquakes and GPS velocity data at Ulith Atoll and Fais Atoll^16^. This fault aligns precisely with the seamount region along the southern rim of the Parece-Vela Basin.

## Sampling details

**Fig. 2:**
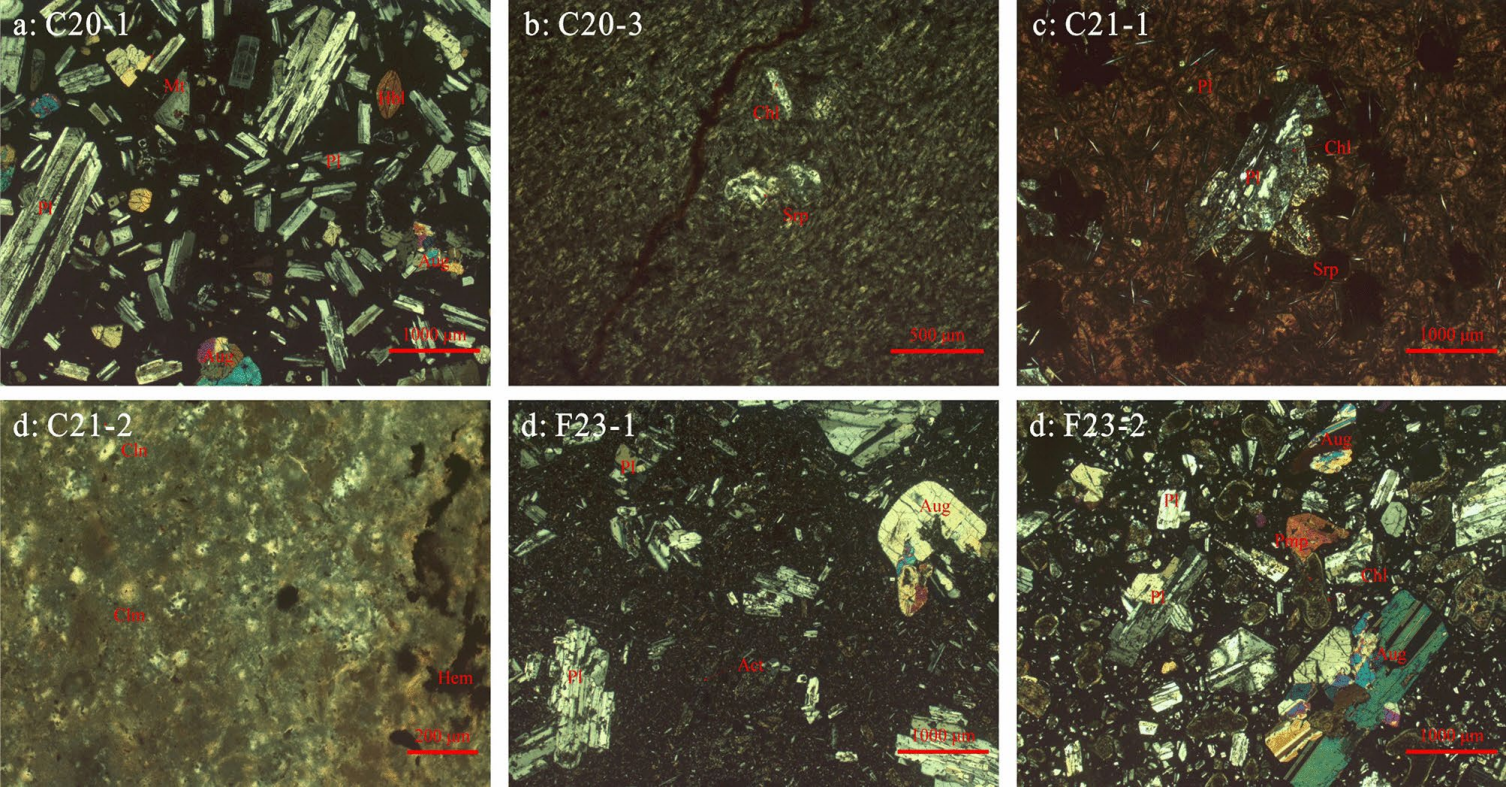
Micro-photographs of samples.

Our samples used for geochemical analyses were taken from south part of the Mariana Arc and Mariana Trough (Fig. 1). C20 corresponds to the shear fault located between Ulith Atoll and Fais Atoll, potentially indicative of upwelling magma through a slab window. East of C20, C21 represents mantle rocks that have been influenced by this upwelling magma. F23 comprises samples collected from the southern segments of the Mariana Trough. C20-1 is basaltic andesite with vitroporphyritic texture that phenocrysts are composed of plagioclase, pyroxene, hornblende, and magnetite (Fig. 2). Both of C20-3 and C21-1 are olive basalt with intersertal texture and oligoporphyritic texture. The groundmass accounts for over 95%, and phenocrysts are mostly plagioclase and serpentinization or chloritization olivine (Fig. 2). C21-2 is basic crystal debris tuff with tuff structure. The debirs are mainly pyroxene, olivine, and plagioclase (Fig. 2). Two sample from F23 (F23-1, F23-2) are basaltic andesite with vitroporphyritic texture (Fig. 2). Phenocrysts are mostly plagioclase, pyroxene, hornblende.

### Analytical methods

Whole-rock major elements were determined on fused glass disks using a ZSXPrimusII X-ray fluorescence spectrometer at the Guangxi Key Laboratory of Exploration for Hidden Metallic Ore Deposits, Guilin University of Technology, Guilin. Analytical precision is better than 2–5%. Trace element abundances were measured on an Agilent7500cx Inductively Coupled Plasma-Mass Spectrometry (ICP-Ms) after complete dissolution. Analytical precision is better than 5–10%, estimated from repeated analyses of the granodiorite standard GSR-9 and kimberlite standard GSR-17.

### Major and trace elements compositions

**Table 1:** Sample composition of major elements (wt%) and trace elements ($\times$10^−6)^.

Sample	C20-1	C20-3	C21-1	C21-2	F23-1	F23-2
longitude	$140.20^{\circ }$E	$140.20^{\circ }$E	$140.61^{\circ }$E	$140.61^{\circ }$E	$141.40^{\circ }$E	$141.40^{\circ }$E
latitude	$11.19^{\circ }$N	$11.19^{\circ }$N	$11.19^{\circ }$N	$11.19^{\circ }$N	$12.39^{\circ }$N	$12.39^{\circ }$N
Al2O3	18.205	15.059	4.632	12.893	19.658	15.479
CaO	7.302	1.751	1.096	3.953	9.666	9.378
Fe2O3	6.573	11.404	8.136	8.444	8.638	9.034
K2O	1.152	0.129	1.871	2.180	0.864	0.648
MgO	4.413	11.928	2.395	8.300	6.658	7.512
MnO	0.132	0.102	0.134	0.155	0.096	0.155
Na2O	2.066	3.498	0.687	2.187	1.787	1.490
P2O5	0.347	0.083	0.185	0.127	0.405	0.188
SiO2	56.721	49.442	77.865	53.814	49.200	50.761
TiO2	0.423	1.458	0.105	0.472	0.757	0.508
LOI	1.736	5.122	2.465	6.631	1.947	3.887
Ba	705.703	3.603	11.952	52.066	30.013	292.456
Rb	24.052	1.392	4.453	25.187	5.662	4.356
Th	6.485	0.130	0.119	1.808	0.199	0.145
Nb	27.085	1.053	0.792	4.789	0.977	0.572
U	1.303	0.043	0.034	0.445	0.072	0.056
Ta	2.591	0.069	0.134	0.285	0.128	0.035
La	46.401	2.963	1.477	10.493	6.307	2.106
Ce	76.861	9.407	3.699	30.330	11.725	4.284
Pr	8.140	1.758	0.663	2.467	1.877	0.797
Sr	756.804	41.196	88.146	201.188	215.549	165.764
Pb	10.297	0.138	1.949	4.281	1.404	0.775
Nd	26.967	10.094	3.309	9.679	9.170	4.291
Sm	4.154	4.180	1.179	2.180	2.640	1.457
Zr	101.210	80.871	32.329	48.295	37.998	22.316
Hf	2.689	2.056	0.798	1.267	1.057	0.631
Eu	1.442	1.622	0.484	0.652	0.983	0.663
Gd	4.527	5.531	1.661	2.485	3.549	1.929
Tb	0.544	1.168	0.343	0.394	0.618	0.350
Dy	2.668	8.562	2.542	2.521	4.053	2.465
Ho	0.584	1.927	0.575	0.541	0.910	0.552
Y	15.627	47.262	15.628	15.697	30.734	17.944
Er	1.707	5.576	1.674	1.577	2.703	1.629
Tm	0.271	0.845	0.255	0.229	0.374	0.238
Yb	1.751	5.404	1.710	1.534	2.583	1.620
Lu	0.288	0.777	0.263	0.221	0.394	0.248

**Fig. 3:**
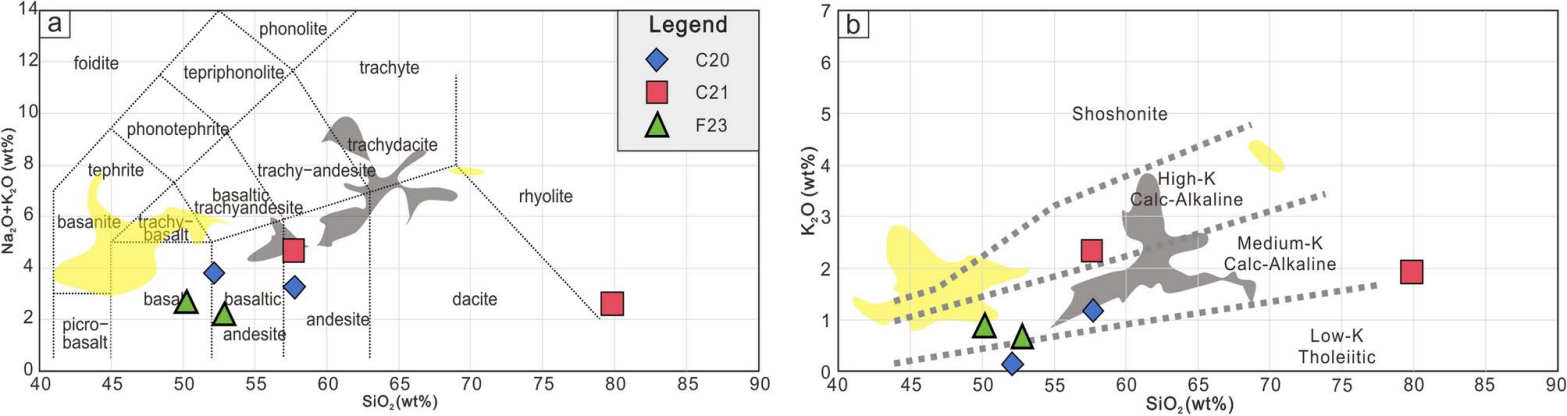
Major elements diagrams. a; TAS diagram (K_2_O+ Na_2_O vs SiO_2_) modified from Maitre, et al.^18^; b: K_2_O vs SiO_2_modified from Peccerillo and Taylor^19^. Yellow zone: data from eastern Pontides, NE Turkey^20^; Gray zone: data from Meseta Chile chico, southern Patagonia, Chile^21^.

Bulk rock major and trace element compositions are reported in Table 1. Loss on ignition (LOI) values of the samples range from 1.74 to 6.63 wt. %, which are due to variable amounts of secondary hydrous/altered minerals. After major oxides analyses recalculated to 100% based on LOI , all samples were plotted on a plot of total alkalis (Na_2_O+K_2_O) versus silica (SiO_2_)^14^. Samples plot into the felds of rhyolite, andesite, basaltic andesite and basalt (Fig. 3). These samples contain intermediate SiO_2_ (46.9–56.7 wt.%) and high Al_2_O_3_ (11.5–19.6 wt.%) beside C21-1 with a high content of SiO_2_ (78.8 wt.%) high Al_2_O_3_ (4.63 wt.%). In addition C21-1 has a relative high FeO/MgO ratio and K_2_O/Na_2_O ratio (Fig. 3).

**Fig. 4:**
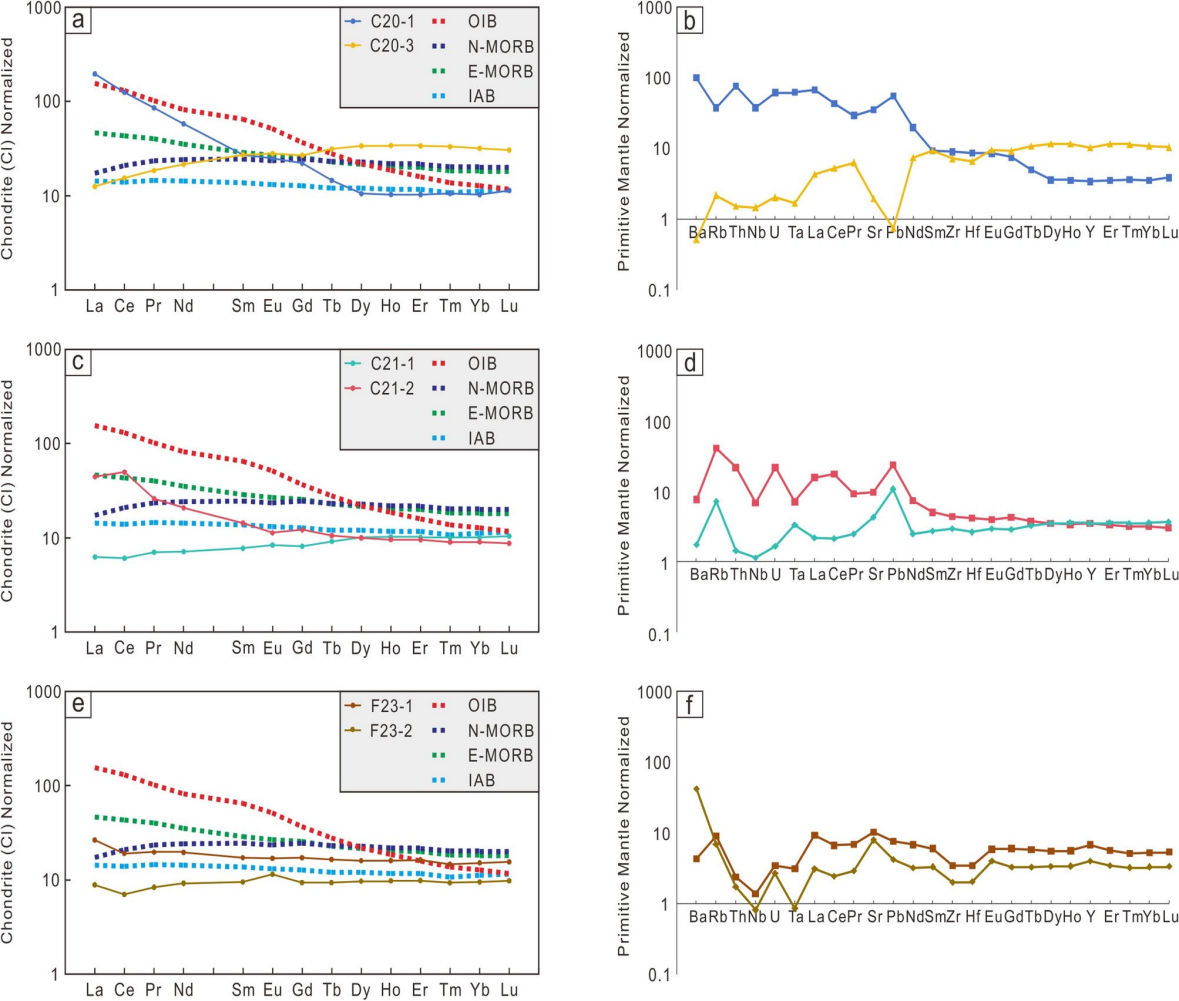
Primitive mantle (trace element; a, c, e) and C1-chondrite (REE; b, d, f) normalized diagrams. OIB = ocean island basalt; N-MORB = normal, depleted mantle-derived mid-ocean ridge basalt; E-MORB = enriched mid-ocean ridge basalt. composition of OIB, N-MORB, E-MORB and normalization values from Sun and McDonough^22^.

The rare earth elements (REE) are significantly fractionated in C20 and C21 (Fig. 4). C20-1 and C21-2 are characterized by high light REE (LREE) abundances. While C20-3 and C21-1 show a low LREE depletement (Fig. 3). In addition, there is no marked Eu anomaly, and slight depletions of Nb and Ta. C21-1, C21-2 and C20-1 show a slight positive Ba anomaly, but C20-3 has a strong negative Pb anomaly. There is an obvious Sr anomaly in F23, But no obvious Pb anomaly.

## Discussion

### Magma process of slab window

**Fig. 5:**
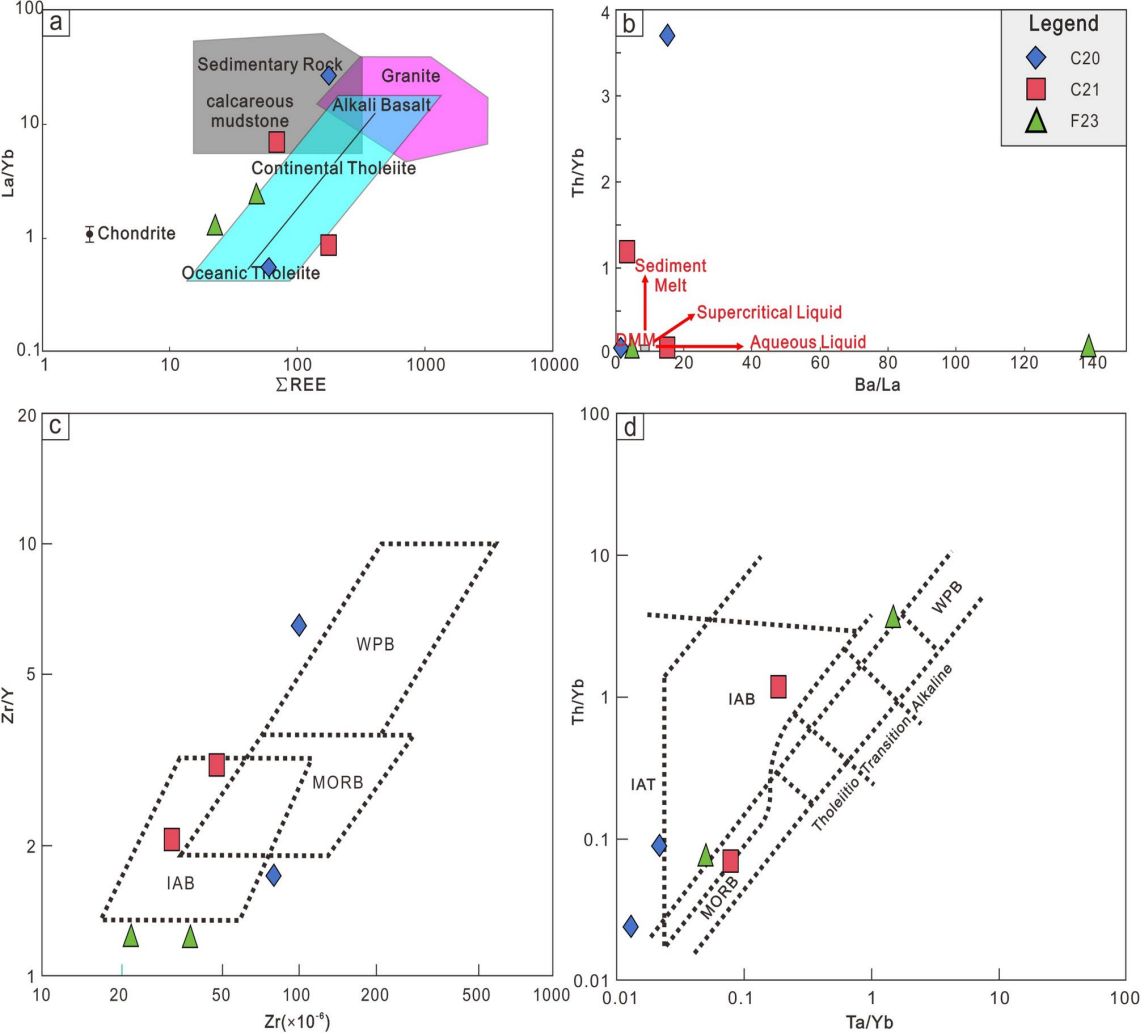
Tectonic discrimination diagrams. a: La/Yb vs total rare earth element diagram modified from Allègre and Minster^39^; b: Th/Yb vs Ba/La diagram modified from Woodhead, et al.^40^; c: Zr/Y vs Zr diagram modified from Pearce and Norry^41^; d: Th/Yb vs Ta/Yb diagram modified from Whattam and Hewins^42^. (WPB: within-plate basalt, MORB: mid-ocean ridge basalt, IAB: island arc basalt; IAT: island arc tholeiite).

The slab window, a region beneath a convergent plate margin largely devoid of subducted slab material, is a significant tectonic feature with implications for mantle dynamics and magmatism. Direct interactions between sub-slab mantle and above-slab mantle through the slab windows^9,23,24^would lead to intense magmatic activity^25–27^. Upwelling of the mantle beneath the subducting slab along the slab window would form a high heat flow environment, which leads to the melting of overlying crustal rocks to form intermediate acidity magma^4,28^, and partial melting of the oceanic crust at the edge of the slab window would produce adakite magma^26,29–31^. The overlying basic crust undergoes partial melting to form adakite rocks^32^, and the upwelling mantle undergoes depressurization and melting to form basic magma. The geochemical signatures of our samples provide key insights into the origin and evolution of the slab window (Fig. 5).

**Fig. 6:**
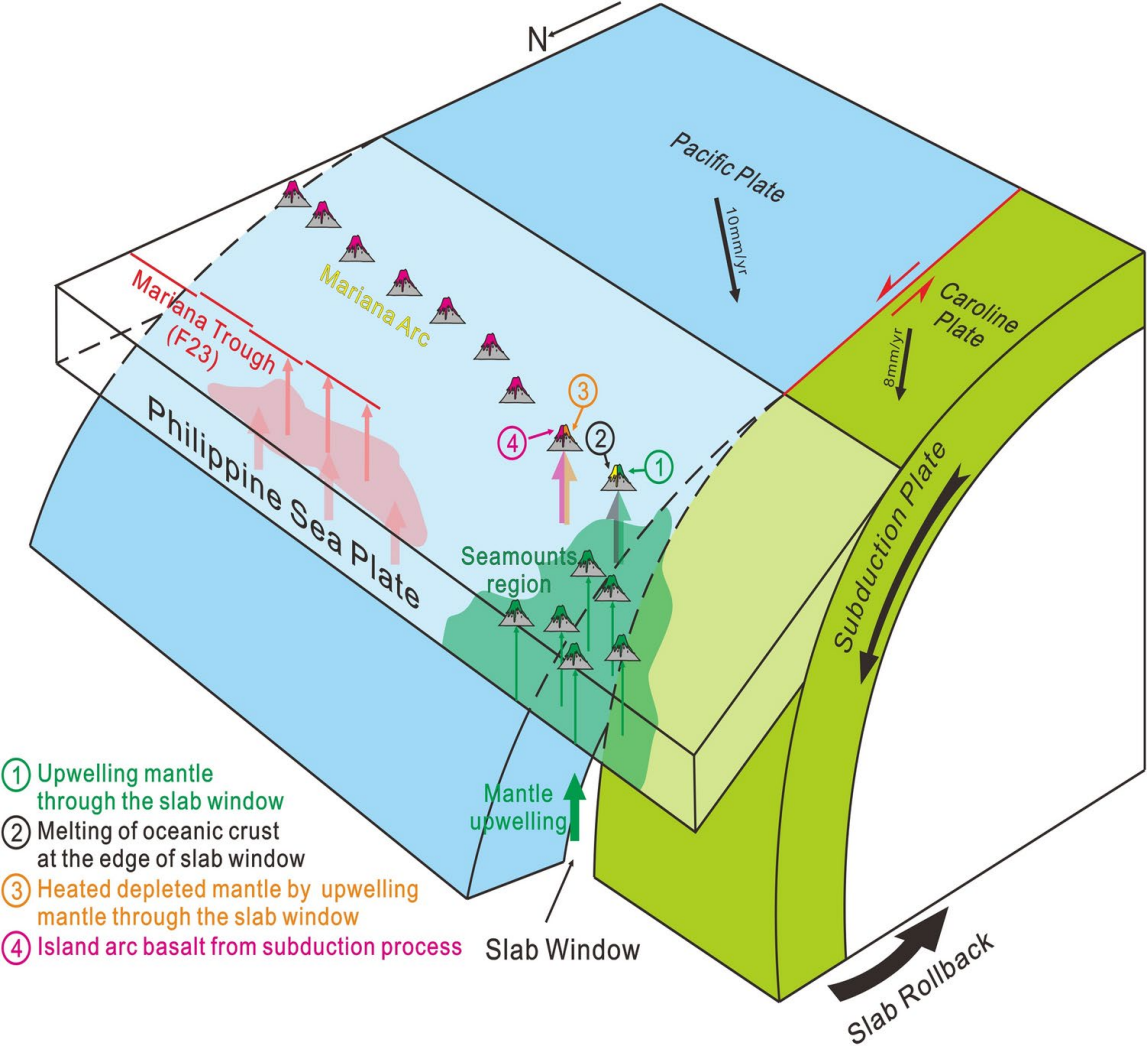
Slab window at the south rim of the Parece-Vela Basin.

C20 is situated in the Mariana Island Arc region and corresponds to a shear fault positioned between Ulith Atoll and Fais Atoll (Fig. 1). C20-1, characterized by a high Th/Ta ratio and enrichment in light rare earth elements (LREE) and large ion lithophile elements (LILE, Fig. 5), bears a strong resemblance to sediment melts within the subduction zone. The geochemistry data of C20-1 exhibits typical adakite characteristics, including SiO_2_>56%, Al_2_O_3_>15%, depleted heavy rare earth elements (HREE) and Y (Yb$<1.9\times$10^−6^,Y$<18\times$10^−6^), high Sr ($>400\times$10^−6^, La/Yb>10) and Sr/Y (>20–40)^33^. Another sample from site C20, labeled C20-3, displays characteristics of low-K tholeiite, specifically high MgO content, low K_2_O, and depleted LREE (Fig. 4)^34,35^. The low-K tholeiitic basalts are thought to be derived from the mantle undergoing some degree of differentiation or interaction with the crust^34–37^. Experimental studies have shown that the low-K tholeiitic basalts can be produced by the melting of hydrous, high-aluminum basaltic compositions, which may be representative of the lower crust in arc settings^35,36^. C20-3 has high content of Al_2_O_3_, which reveals the differentiation or interaction with the lower crust. But the low Th/Yb ratio and Ba/La ratio observed in C20-3 (Fig. 5) indicate that it is not influenced by the common fluid signatures present in the island arc region. In addition, C20-3 does not display the negative Ta and Nb anomaly typically found in the island arc region. Hence, C20-3 may represent the magma of the upwelling mantle through the slab window unaffected by subduction. And the high content of Al_2_O_3_ may be the result of lower crust of the subducted slab instead of the overlapping crust. By combining the characteristics of C20-1 and C20-3, it is proposed that the magma source of C20 could originate from the edge of a slab window, where adakite rocks (C20-1) form from the melting of oceanic crust at the edge of slab window, and tholeiite (C20-3) is generated by the upwelling mantle passing through the slab window (Fig. 6).

C21 is also situated within the Mariana Island Arc region (Fig. 1). While C21 lies east of C20 and is not directly positioned over the slab window, samples taken from C21 still demonstrate the impact of the slab window. C21-1 exhibits geochemical characteristics similar to mid-ocean ridge basalt (MORB), suggesting a depleted mantle origin under high heat conditions, as proposed by Zhang, et al.^38^. And similar to C20-3, C21-1 does not exhibit a negative anomaly of Ta and Nb. C21-2, another sample from C21, exhibits pronounced Nb-Ta anomalies, higher total rare earth element content and Th/Ba ratio (Fig. 5), indicating its origin as an island arc basalt (IAB). The hot upwelling of the mantle beneath the subducting slab at the slab window^4^ would spread outwards, heating the depleted mantle, and this heated mantle serves as the magma source for C21-1. Additionally, the ongoing subduction process continuously generates island arc basalt (IAB) magma, represented by C21-2 (Fig. 6).

### Regional influence from the slab window

The intraplate volcanism observed in the seamounts region at the south rim of the Parece-Vela Basin is likely related to the presence of the slab window. Several factors could contribute to the anomalous mantle conditions necessary for such volcanism, including the westward-extending spreading center of the Mariana Trough, rear-arc volcanism, or the slab window. Based on the Slab2 model^43^and the estimated slab depths within the seamount region, we can exclude rear-arc volcanism as the primary driver, as it typically originates from slab melting at depths greater than 140 km^44^. Compared to the spreading center at east of Palau Island^45^, the westward extending spreading center of the Mariana Trough may not lead to the seamount region. Thorkelson^1^ defined three types of slab window volcanoes: 1) volcanoes in the forearc resulting from the “blow-torch effect,” 2) volcanoes located above slab edges caused by the partial melting of subducted oceanic crust, and 3) volcanoes situated above the slab window formed in response to the decompression-melting of upwelling sub-slab mantle. The second type of slab window volcanoes has been identified in C20. Additionally, the intraplate volcanism noted in the seamounts region at the southern edge of the Parece-Vela Basin, located directly above the slab window, may represent the third type of slab window volcanoes. These volcanoes are likely formed due to the upwelling and decompression-melting of the sub-slab mantle.

The southernmost section of the Mariana Trough is particularly noteworthy, as the samples from this region (F23-1 and F23-2) exhibit IAB-like characteristics and low Th/Ba ratios indicative of aqueous fluid influence. C21-1 originates from a depleted mantle that has been subjected to high heat conditions, a direct consequence of the presence of the slab window. When compared to C21-1, samples from F23 exhibit similar trace element characteristics (Fig. 4). However, a higher Ba/La ratio and lower Zr content observed in F23 samples (Fig. 5) suggest a more pronounced influence from the subduction process at this location. Based on the Slab2 model^43^, the corresponding slab depth of the spreading center segment ranges from 40 km to 60 km which is even shallower than the Izu-Bonin-Mariana island arc region^43^. The depth is so shallow that the magma supply from the subduction process may be limited. Tomography studies have revealed a low-velocity anomaly and crustal thinning in this region, suggesting that it could represent an incipient spreading center^46^. Numbers of tension earthquakes (Fig. 1) suggest that this spreading center segment is quite active. Therefore, the slab window may be extra factors to produce additional magma here. The subducted slab would release fluids^15^ into the above-slab mantle. As the sub-slab mantle rises through the slab window, it brings hotter and drier conditions compared to above-slab mantle. This upwelling provides additional heat, similar to the process observed at C21-1. These two factors including the fluids from the subducted slab and the hotter conditions brought by the upwelling sub-slab mantle through the slab window, would induce significant partial melting within the mantle wedge, ultimately giving rise to an active back-arc spreading center.

The anomalously high heat flow is an important feature for identifying a slab window^1^. For example, the slab gap in the western Anatolia has been well defined^47^. A mean value of heat flow for the western part of Anatolia of 107${\pm }$45 mWm^−2^ (2.55 HFU) has been obtained, and the heat flow anomaly in the central part of western Anatolia related to the slab gap is about 150 mWm^−2^(3.58HFU^48^) . Watanabe, et al.^49^ published a heat flow data of over 3.5 HFU at the northwest corner of the seamounts region at the south rim of the Parece-Vela Basin. And the site is also located near the fossial spreading center of the Parece-Vela Basin. But this spreading center may be not the reason for this high heat flow because the spreading of the Pareve-Vela basin has ceased since $\tilde{1}$5 Ma^50,51^. Though the heat flow in the the Parece-Vela Basin is relatve high (over 2.0 HFU^52^), the 3.5 HFU at the northwest corner of the seamounts region (Fig. 1) is quite high which is even higher than the heat flow in the Godzilla Megamullion oceanic core complex region^53^ at north. Therefore, this anomalously high heat flow data may be caused by the slab window.

### Geometry and dynamics of the slab window

**Fig. 7:**
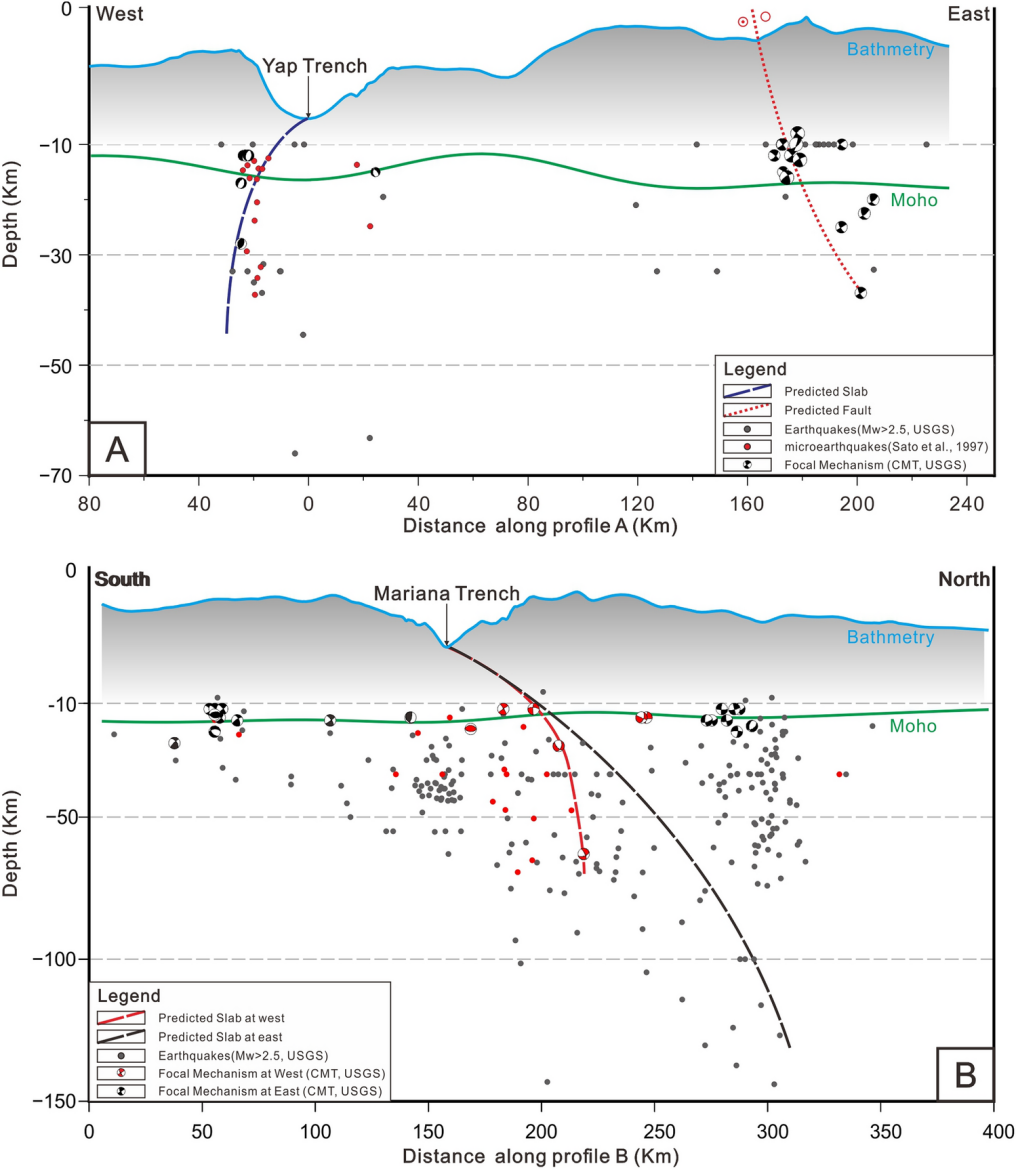
A: Predicted shearing fault^12^; B: Predicted slab of the subducted Pacific Plate (Black dashed line) and Caroline Plate (Red dashed line), data source as Figure 1. Location of profiles are shown as purple lines in Figure 1.

Subduction roll-back of Mariana Trench driven by gravity of subducted slab has been consensus now^54^. Under this view, the subducted slab at Mariana Trench may be under great tension horizontally considering the arc-shaped Mariana Trench, because the surface area of subducted slab is bigger after subduction. The arc-shaped geometry of the trench and the concentration of stress at its ends make the slab particularly vulnerable to tearing. Earthquakes suggested that the shear fault between Ulithi Atoll and Fais Atoll had cut through the lithosphere^12^, because the earthquakes associated with the shearing fault (Fig. 7A) are distributed at depths of approximately 10–40 km, which corresponds to the depth of the oceanic lithosphere. The horizontal tension and different subduction angle may lead to tearing of subducted slab along this shear fault.

Subduction zone earthquakes are usually distributed at the interface between the subducting and overriding plates, or inside the sinking plate (slab)^55^. But earthquakes in the seamounts region is clearly less than that in the normal Mariana subduction zone (Fig. 1), which may represent absent subducting slab here. Typically, thrusting earthquakes, which are related to the subduction process, dominate in the back-arc region. However, earthquakes in the seamount region are primarily of the strike-slip type, which may be caused by the shearing fault within the subducted slab. Due to the lack of suitable tomography in this region, subducted slab at both sides of the shearing fault was predicted by the distribution of earthquakes in the purple dotted box (Fig. 1) with the boundary of the extended line along the shearing fault (Profile B in Fig. 1). The subduction angle at west is greater than that at east (Fig. 7B), indicating that the subducted slab has been torn along the shearing fault, which is the slab window.

The formation of the seamounts region at the southern edge of the Parece-Vela Basin is closely linked to the presence of the slab window. While the spreading center of the Parece-Vela Basin is located in close proximity to the slab window, there has been no notable acceleration in its spreading rate^51^. Therefore, it is reasonable to hypothesize that the slab window formed after the cessation of spreading activities in the Parece-Vela Basin ($\tilde{1}$5 Ma^56^) or later.

## Conclusion

The shearing fault between the Ulithi Atoll and Fais Atoll cuts through the lithosphere. Subduction of this cut Pacific slab along the Mariana Trench causes a slab window beneath the south rim of the Parece-Vela Basin. Four samples describe the magma process of the slab window. At the edge of the slab window, upwelling mantle through the slab window (C20-3) and melting of oceanic crust (C20-1) are two magma sources. At a slightly away position from the slab window, the upwelling mantle through the slab window heats up the depleted mantle (C21-1) to produce the magma, besides that the continuous subduction process produces common IAB magma at the island arc region (C21-2). Shape of the slab window can by roughly modeled by the distribution of earthquakes, but further tomography work is necessary.

## Data Availability

Data is provided within the manuscript.
